# Brugada Pattern in a Child with Severe SARS-CoV-2 Related Multisystem Inflammatory Syndrome

**DOI:** 10.3390/pediatric13030058

**Published:** 2021-09-01

**Authors:** Angelica De Nigris, Angela Pepe, Giangiacomo Di Nardo, Antonietta Giannattasio, Annamaria Pagano, Carolina D’Anna, Stefania Muzzica, Selvaggia Lenta, Giovanni Maria Di Marco, Vincenzo Tipo

**Affiliations:** 1 Department of Woman, Child and General and Specialist Surgery, University of Campania “Luigi Vanvitelli”, 80138 Naples, Italy; 2 Department of Medicine, Surgery and Dentistry “Scuola Medica Salernitana”, Pediatrics Section, University of Salerno, 84081 Baronissi, Italy; angpepe01@gmail.com; 3 Division of Cardiology, Department of Pediatrics, Santobono-Pausilipon Children Medical Hospital, 80129 Naples, Italy; gg.dinardo@libero.it (G.D.N.); giovanni.m.dimarco@gmail.com (G.M.D.M.); 4 Pediatric Emergency and Short Stay Unit, Santobono-Pausilipon Children’s Hospital, 80129 Naples, Italy; antonella.giannattasio@virgilio.it (A.G.); dannacarol@alice.it (C.D.); stefaniamuzzica@hotmail.it (S.M.); selvylenta@hotmail.com (S.L.); enzotipo@libero.it (V.T.); 5 Department of Translational Medical Science, Section of Pediatrics, University of Naples “Federico II”, 80126 Naples, Italy; annamaria16392@gmail.com

**Keywords:** SARS-CoV-2, children, multisystem inflammatory syndrome, Brugada syndrome

## Abstract

This report presents the first case of Brugada pattern complicated by a supraventricular arrhythmia in a child with SARS-CoV-2 related Multisystem Inflammatory Syndrome in Children (MIS-C). A 7-year-old boy came to our Emergency Department with 7 days of abdominal pain and fever. MIS-C was diagnosed on the basis of the clinical, laboratory and instrumental tests. On admission, ECG showed type 1 Brugada pattern in the right precordial leads. During hospitalization the onset of supraventricular arrhythmias complicated the clinical picture. This case underlines management complexity of supraventricular arrhythmic events, different from atrial fibrillation, in patients with Brugada pattern in the context of a systemic inflammatory condition with significant cardiac involvement. All potential therapeutic choices should be considered to ensure the best outcomes.

## 1. Introduction

Since April 2020, a novel systemic inflammatory condition temporally associated with severe acute respiratory syndrome coronavirus 2 (SARS-CoV-2) named multisystem inflammatory syndrome in children (MIS-C) has been described [1]. This syndrome is characterized by fever, conjunctival injections, rash, gastrointestinal symptoms and cardiovascular complications [2] in a patient <21 years positive for current or recent SARS-CoV-2 infection by RT-PCR, serology, or antigen test [3].

Currently, several case reports described adult patients with Brugada pattern in the setting of COVID-19 [4,5,6,7,8,9,10].

Choi et al. [11] described the first case of Brugada pattern in an adolescent tested positive for SARS-CoV-2. In this case Brugada pattern was detected in the acute phase of the disease and it resolved along fever disappearance and improvement of inflammatory markers.

A case of Brugada pattern in a child with a post-infectious SARS-CoV-2 related disease has been recently reported [12]. In addition to this, our case highlights management complexity of supraventricular arrhythmic events, different from atrial fibrillation, in the context of a systemic inflammatory condition with significant cardiac involvement and underlying proarrhythmic condition.

## 2. Case Report

A 7-year-old male presented to the pediatric Emergency Room with 7 days of fever, abdominal pain and vomiting.

**Figure 1:**
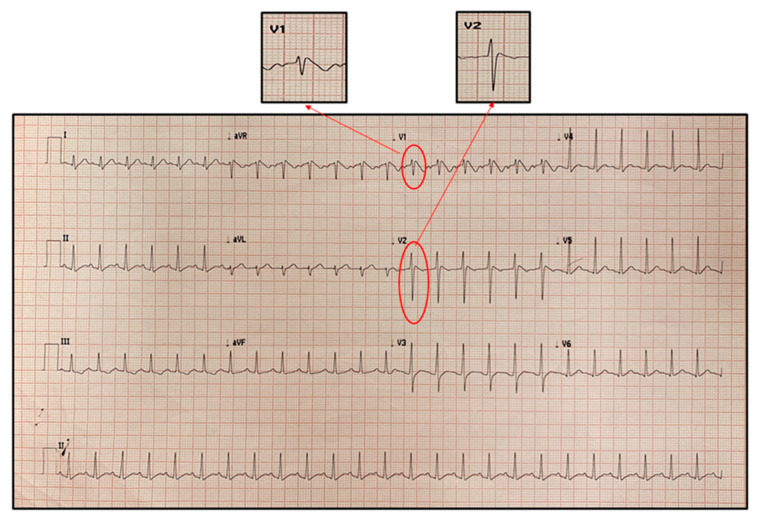
First electrocardiogram recorded on admission showing Brugada type 1 pattern in V1 and V2. It is characterized by a coved ST-segment elevation displaying J-point amplitude or ST-segment elevation ≥2 mm, followed by a negative T wave in more than one right precordial leads (V1 to V3).

At admission he presented poor general conditions, with body temperature of 38.3 °C, oxygen saturation in room air of 99%, respiratory rate of 26 breaths/min, pulse rate of 150 beats/min and systemic blood pressure of 95/55 mmHg. On physical examination he was pale with bilateral conjunctival hyperemia and periorbital edema; he presented tense and painful abdomen with signs of defense in the periumbilical area; no lymphadenopathy or cutaneous rash were detected. At entry, electrocardiogram (ECG) revealed type 1 Brugada pattern in the right precordial leads (Figure 1).

**Figure 2:**
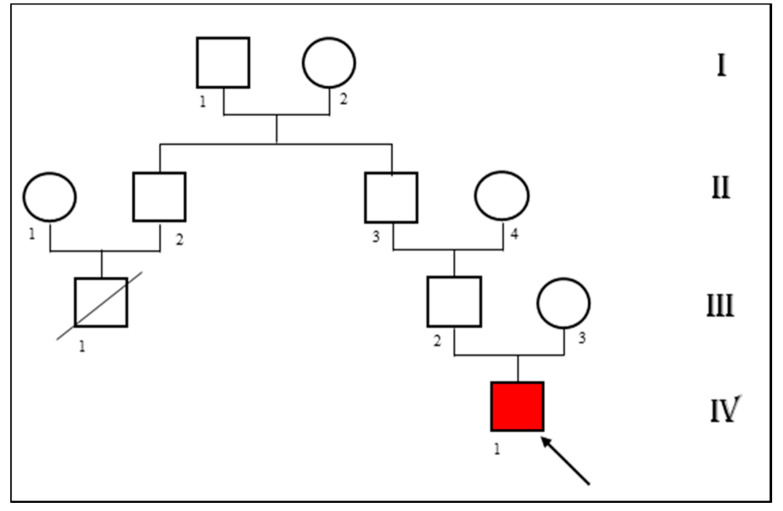
Pedigree showing the patient’s (IV 1) family history. A cousin of the patient’s father died of sudden cardiac death at the age of 35 (III 1).

Patient’s medical history was unremarkable except for paucisymptomatic SARS-CoV-2 infection over two months prior the hospital admission. Personal history was negative for syncope or other cardiac manifestations. We asked the parents for a previous ECG of the child. The patient had one at the age of five. The mother reported that it was normal but never found and exhibited it. He had no familiarity for congenital heart disease. The patient’s father referred a suspicious sudden cardiac death at young age in his family (a cousin dead at age of 35 years old) (Figure 2). Unfortunately he also reported that an autopsy was not carried out to reveal the precise causes.

**Figure 3:**
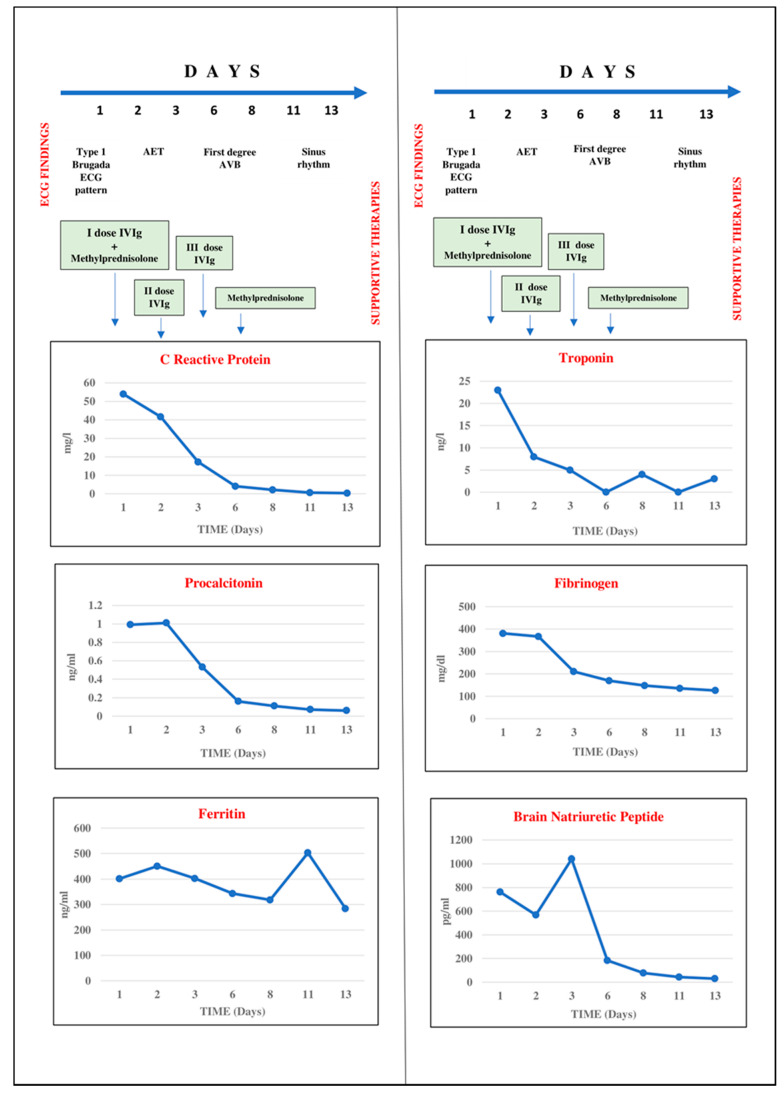
Progressive improvement of inflammation and cardiac markers and ECG normalization during treatment with intravenous immunoglobulins and methylprednisolone boluses. Troponin on admission was 23 ng/L (normal reference value for age < 14) and normalized at the 2nd day of treatment.

Laboratory tests revealed white blood cell count of 16,700/µL (neutrophils 85.2%, lymphocytes 10.8%), Hb 12.1 g/dL, platelets 248,000/µL, serum Na^+^ 130 mEq/L, K^+^ 3.5 mEq/L, albumin 2.6 g/dL, procalcitonin 0.99 ng/mL (normal reference value < 0.5), C-reactive protein 53.9 mg/L (normal reference value < 5), fibrinogen 381 mg/dL (normal reference value 180–400), D-dimer 636 ng/mL (normal reference value < 270), ferritin 452 ng/mL (normal value for sex and age < 150), interleukin-6 fourteen times upper the normal reference value, triglycerides 164 mg/dL, troponin 23 ng/L (normal reference value for age < 14), brain natriuretic peptide 763 pg/mL (normal reference value < 100) (Figure 3). Liver and renal function tests were within normal range.

**Figure 4:**
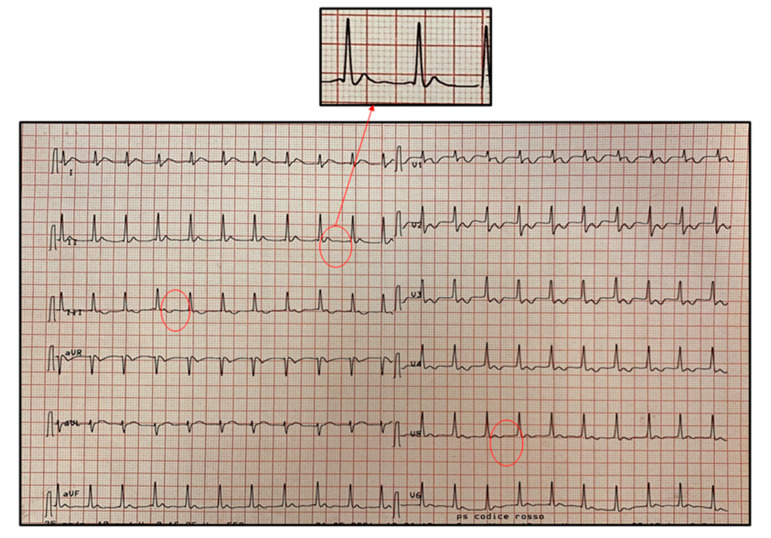
Atrial ectopic tachycardia (AET) at heart rate of 120 beats/min recorded on day 2 of hospitalization. The diagnosis is based on the presence of a regular narrow QRS complex tachycardia with an abnormal P-wave morphology originating from an ectopic focus. As shown in the magnification insert, the P-wave is not visible because it is morphologically different from the sinus one and it is fused with the preceding T-wave.

Nasopharyngeal swab for SARS-CoV-2 resulted negative. Anti-SARS-CoV-2 IgG antibodies were found positive, with absence of specific IgM. Ultrasonography evaluation of the abdomen was normal except for ileal thickening. Echocardiography showed an ejection fraction (EF) of 50% with interventricular septum hypokinesia and moderate pericardial effusion. Followed with serial ECGs, his Brugada pattern resolved by day 1 of hospitalization along with fever resolution. It is to note that at this time serum inflammatory markers were still elevated (see Figure 3). On the 2nd day of hospitalization, ECG revealed atrial ectopic tachycardia (AET) at heart rate of 120 beats/min (Figure 4) in conditions of weak hemodynamic balance.

Differential diagnosis considering patient’s clinical presentation and his past medical history included: MIS-C, Kawasaki disease, Kawasaki disease shock syndrome and toxic shock syndrome. On the basis of clinical, laboratoristic and imaging evaluation, patient received diagnosis of MIS-C. He underwent medical treatment with intravenous immunoglobulins (IVIg) (2 g/kg single infusion) and intravenous bolus of methylprednisolone (30 mg/kg/day for 3 consecutive days) followed by intravenous methylprednisolone at a dosage di 2 mg/kg/day, according to the current guidelines [3].

**Figure 5:**
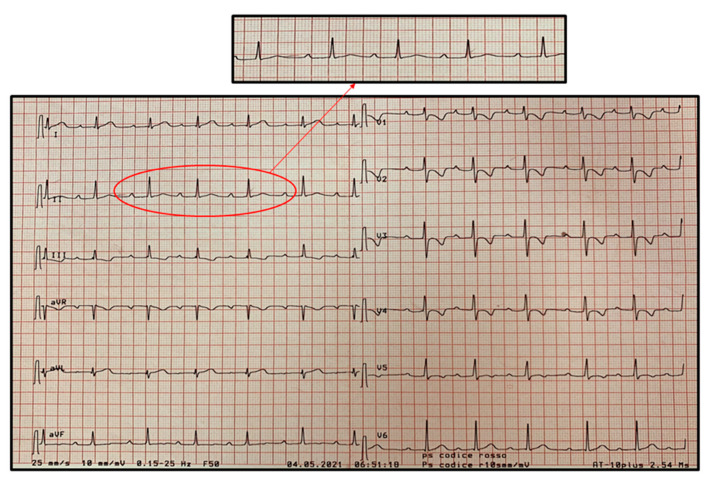
Sinus rhythm with first-degree atrioventricular block (AVB) in progress of MIS-C associated with coronavirus disease 2019 (COVID-19). First-degree AVB is a delay, without interruption, in conduction from atria to ventricles resulting in an increased PR interval greater than 200 ms.

**Figure 6:**
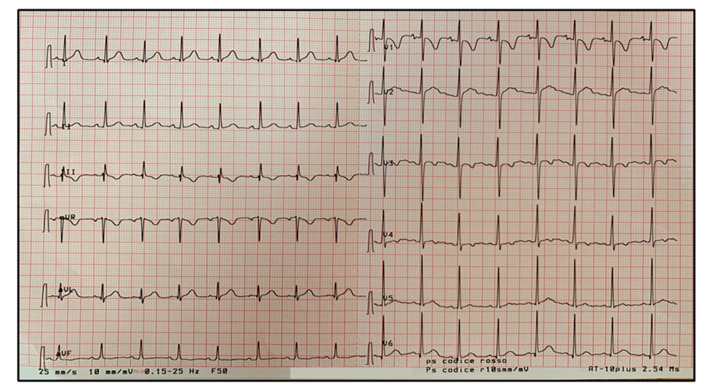
Complete ECG normalization after 7 days of MIS-C treatment according to current guidelines.

Synchronized direct current cardioversion (a first shock at 75 joule and a second one at 100 joule) was performed to stop AET without success. Therefore, pharmacologic therapy with oral sotalol (5 mg/kg/day) was started under close monitoring of vital signs and ECG-trace, with resolution of the arrhythmia and subsequent evidence of sinus rhythm and first-degree atrioventricular block (AVB) (Figure 5). Sotalol was discontinued after 7 days with a complete ECG normalization (Figure 6).

Clinical, laboratory and cardiac imaging improvement was observed, and the child was discharged after 20 days of hospital stay. No recurrence of symptoms or ECG alteration were recorded during the 1-month follow up. After two months, the genetic test for Brugada syndrome (BrS) was negative for pathogenetic mutations in the SCN genes.

## 3. Discussion

BrS is an inherited arrhythmic disease with an increased risk of sudden cardiac death due to potentially fatal ventricular tachy-arrhythmias. It is characterized by a typical ECG framework represented by an incomplete right bundle-branch block and a coved-type ST segment elevation ≥2 mm in the right precordial leads (from V1 to V3), called Brugada pattern type I, that presents spontaneously or after provocation test with sodium channel blockers [13]. However, many patients affected by BrS have different shapes of ST-segment elevation (“saddleback” rather than “coved”), which are suggestive, but not diagnostic (type II or type III Brugada). Inheritance of BrS occurs via an autosomal dominant mode of transmission. The inward sodium or calcium current or an increase in one of the outward potassium currents has been shown to be associated with the BrS phenotype. SCN5A, the gene encoding for the α subunit of the cardiac sodium channel, account for less than 30% of clinically diagnosed BrS patients [14]. It is known that type I Brugada pattern may appear in response to fever, electrolytes disturbances, drugs or medication.

Since April 2020, a novel systemic inflammatory condition temporally associated with SARS-CoV-2 named MIS-C has been described [1].

To our knowledge, this is the second case describing a Brugada pattern in a child with a post-infectious SARS-CoV-2 related disease [12] but the first case further complicated by a supraventricular arrhythmia.

Common cardiac manifestations in MIS-C are ventricular disfunction, coronary anomalies and conduction abnormalities, most commonly tachy-arrhythmia and first degree ABV, especially in patients with depressed EF [15].

Our patient showed a transient Brugada ECG pattern type I, probably unmasked by the cytokine-induced inflammatory cascade of MIS-C, that disappeared along adequate fever treatment. On day 2, ECG revealed AET, a complication often described in patients affected by MIS-C [15]. In consideration of a weak hemodynamic balance, it was initially treated with two attempts of Synchronized Direct Current cardioversion. After unsuccessful electrical cardioversion, restoration of sinus rhythm was attempted with sotalol, an antiarrhythmic drug without absolute contraindication to the use in patients with BrS [13].

This therapy resulted in sinus rhythm restoration and first degree AVB, that maybe potentially caused both by sotalol and evolution of MIS-C, as previously reported [16].

## 4. Conclusions

Our case is clinically significant because it is the first case of Brugada pattern in MIS-C complicated by a supraventricular arrhythmia. It underlines management complexity of arrhythmic events in patients with Brugada pattern in the context of a MIS-C with significant cardiac involvement.

## Data Availability

No additional data sets are associated with this paper.
